# What are the mechanisms of action of anti-inflammatory agents in adipose tissue? A protocol for systematic review and meta-analysis

**DOI:** 10.1097/MD.0000000000024677

**Published:** 2021-02-26

**Authors:** Sara Sayonara da Cruz Nascimento, Jaluza Luana Carvalho de Queiroz, Amanda Fernandes de Medeiros, Ana Clara de França Nunes, Grasiela Piuvezam, Bruna Leal Lima Maciel, Thaís Souza Passos, Ana Heloneida de Araújo Morais

**Affiliations:** a Nutrition Postgraduate Program, Center for Health Sciences; b Biochemistry Postgraduate Program, Biosciences Center; c Postgraduate Program in Public Health; d Department of Public Health, Center for Health Sciences; e Department of Nutrition, Federal University of Rio Grande do Norte, Natal, RN, Brazil.

**Keywords:** adipose tissue, anti-inflammatory agents, body fat, inflammation, obesity, systematic review

## Abstract

**Background:**

Obesity is a disease characterized by the abnormal accumulation of adipose tissue in the body, triggering a chronic subclinical state of inflammation. Bioactive compounds, given their anti-inflammatory properties, are a safe and promising alternative in controlling the inflammatory condition of obesity. This study describes a systematic review protocol aiming to analyze the anti-inflammatory molecules mechanisms and compounds action on adipocytes.

**Methods:**

Preferred Reporting Items for Systematic Review and Meta-Analysis Protocols (PRISMA-P) will outline the protocol and PRISMA to the systematic review. The databases used for research will be PubMed, Science Direct, Scopus, Web of Science, BVS, and EMBASE. Experimental studies performed on rats and mice with a control group that describes treatment with anti-inflammatory agents (drugs, nutraceuticals, bio active compounds, among others) at any frequency, time, and dose will be included. Three independent reviewers will select studies and extract data. The evaluation of the methodological quality of each research will be performed using the SYRCLE tool. If at least 2 studies show clinical and/or methodological and/or statistical homogeneity, a meta-analysis will be performed, using the RevMan Analyzes statistical package in Review Manager v.5.3.

**Results:**

In this study, we hope to find a considerable number of articles presenting mechanisms involved in the action of anti-inflammatory molecules and compounds on adipocytes.

**Conclusion:**

The systematic review produced from this protocol will present evidence on the mechanisms involved in the action of anti-inflammatory molecules and compounds in adipocytes. It will also contribute to developing new research and new insights about anti-inflammatory therapies with a future application view.

**Record of systematic review:**

This review was registered with the International Register of Prospective Systematic Reviews on May 18, 2020 (registration: CRD42020182897). Available at: https://www.crd.york.ac.uk/prospero/display_record.php?ID=CRD42020182897.

## 1 Introduction

According to the World Health Organization (WHO), the number of people with obesity in the world has tripled in the last 20 years, demonstrating significant and constant growth and health complications, such as insulin and leptin resistance, and cardiovascular diseases, among others. Thus, obesity is considered the fifth leading cause of mortality in the world.^[1]^

Obesity is a multifactorial disease characterized as an abnormal accumulation of adipose tissue in the body, triggering a chronic subclinical state of inflammation.^[2]^ In this condition, adipose tissue is responsible for increasing the secretion of inflammatory mediators, such as Tumor Necrosis Factor alpha (TNF-α) and Interleukin-6 (IL-6) that induce various complications in chronic and metabolic diseases.^[3–7]^

Changes in the adipose tissue structure and composition occur with positive energy imbalance, causing its expansion by hypertrophy and/or hyperplasia.^[8,9]^ According to Illesca et al^[10]^, when suffering from hyperplasia, the adipose tissue causes an inflammatory state, which can be improved by molecules and compounds that act on inflammation. These actions may be involved in the transcription factors modulation, such as nuclear factor kappa B (NF-κB), nuclear factor erythroid 2-related factor 2 (Nrf2), sterol regulatory element-binding protein 1 (SREBP-1c), and peroxisome proliferator-activated receptor gamma (PPAR-γ). These compounds may also act in the modulation of the Toll-like receptors (TLR) and AMP-activated protein kinase (AMPK) signaling pathways, reducing adipogenesis and promoting energy expenditure and the transformation of white adipose tissue into brown.^[11,12]^

Even though obesity treatment is primarily directed to changing eating habits and changing lifestyles, medications are used and designed to prevent or control obesity. Many medications are still used and explicitly designed to prevent and control overweight, yet advances in pharmacological therapy are still limited.^[13,14]^ New molecules and compounds have been promising alternatives reducing the problems related to the inflammatory condition of obesity, promoting or not weight loss. Synthetic drugs present several adverse effects, and this fact has driven more research for nutraceuticals as they may be safer alternatives to manage obesity.^[15–17]^ Several studies in the literature address the action of bioactive compounds in adipose tissue, but studies that gather evidence regarding the mechanisms of action already established are not yet reported.^[15–17]^

Given the search for new alternatives for the treatment of obesity and its direct correlation with inflammation, it is necessary to understand what mechanisms are involved in the action of these compounds and molecules on adipose tissue to improve pre-clinical and clinical studies and future perspective on humans.

## 2 Methods and analysis

### 2.1 Protocol and registration

This protocol was registered in the International Prospective Register of Systematic Reviews (PROSPERO) on May 18, 2020, with registration number CRD42020182897, and available at https://www.crd.york.ac.uk/prospero/display_record.php?ID=CRD42020182897.

### 2.2 Analysis plan

**Figure 1:**
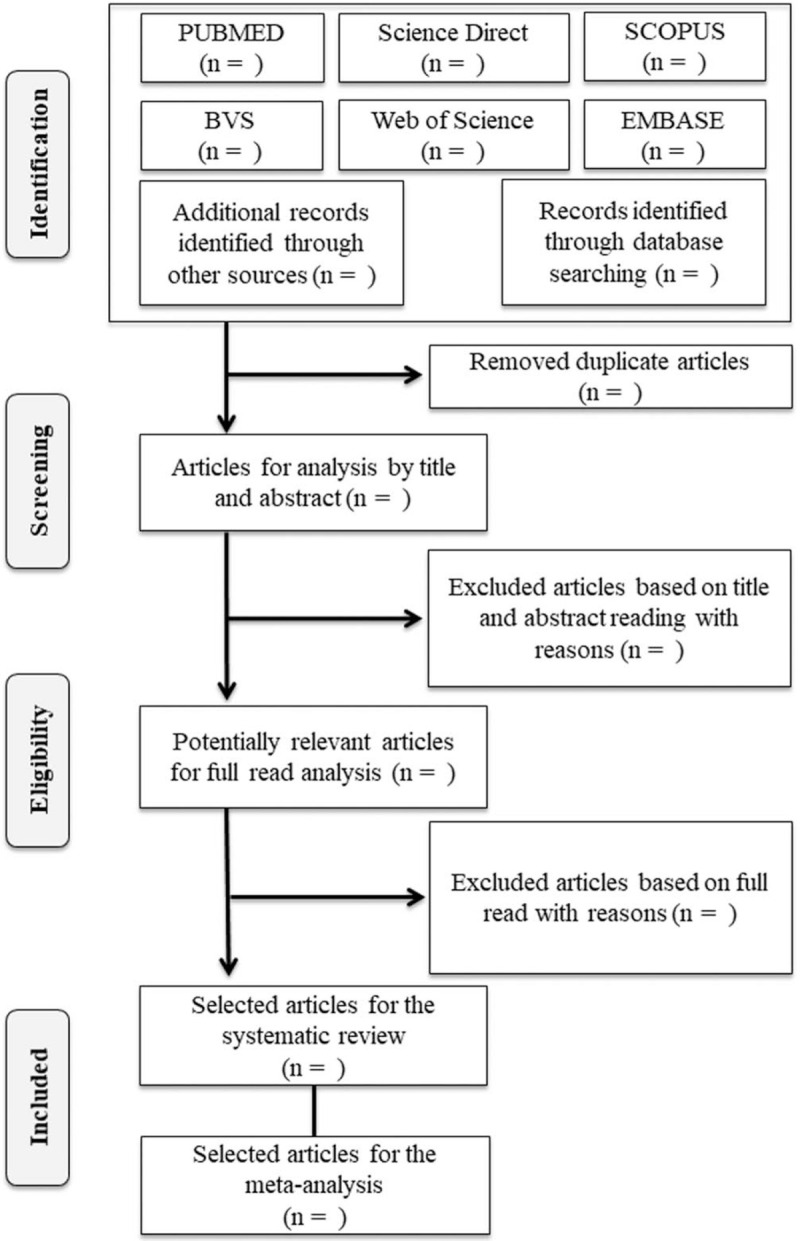
Flowchart for selection of study articles based on PRISMA-P.

This protocol will be developed following the Preferred Reporting Items for Systematic Reviews and Meta-Analyses Protocols (PRISMA-P), and the systematic review according to the Preferred Reporting Items for Systematic Reviews and Meta-Analyses (PRISMA), which provides a selection flow diagram (Fig. 1).

### 2.3 Eligibility criteria

The articles must meet the eligibility criteria according to the PICOS (population, interventions, control, and study results), described in Table 1.

**Table 1:** Elements of the research question according to the PICOS strategy.

Description	Abbreviation	Elements of the question
Population	P	Overweight and/or obese male rats and/or mice
Interventions	I	Treatment with anti-inflammatory agents (Medicines, nutraceuticals, bioactive compounds, among others)
Control or comparison	C	Overweight and/or obese or eutrophic male rats and/or mice without treatment
Outcomes	O	Effect on adipose tissue (mechanism of action
Types of studies	S	In vivo experimental studies

PICOS = problem, intervention, control, outcomes and study results.

### 2.4 Inclusion criteria

This review will include Original articles resulting from experimental studies performed on rats and mice with a control group, that represents the treatment with anti-inflammatory agents (medicines, nutraceuticals, bio active compounds, among others) in any frequency, time and doses.

#### 2.4.1 Exclusion criteria

Documents that are not scientific articles, case reports, reviews, studies with other animal models, studies that do not describe the mechanism of action, time of the experiment, frequency, and doses administered and studies without a control group.

#### 2.4.2 Search strategy

Searches will be conducted in the following databases: PubMed; Science Direct; Scopus; Web of Science; BVS and EMBASE by 3 independent researchers (S.S.C.N., J.L.C.Q., and A.F.M.), using the complete search strategy based on the “animal” and “article” search components (Table 2). No publication date or language restrictions will be applied.

**Table 2:** Search strategy for each database.

Database	Search Strategy
PUBMED	Inflammation and “adipose tissue” and “Anti-inflammatory agents” and obesity
SCOPUS	
SCIENCE DIRECT	
WEB OF SCIENCE	
BVS	
EMBASE	

Initially, the duplicates will be removed from the database, after that the title and summary will be read. If the 3independent researchers disagree on the inclusion of any study in the review, a fourth researcher (A.C.F.N.) will decide whether or not to include the research. Studies excluded will be recorded, and their reason for exclusion will be reported in the review. All researchers will then review the full text of all studies considered eligible for inclusion. A specific application will be used to conduct systematic reviews (Rayyan QCRI)^[18]^, and for the management of references will be applied to the software Mendeley.^[19]^

#### 2.4.3 Types of results

The primary outcomes include the identification of the effect of treatment with anti-inflammatory agents on adipose tissue, as well as the mechanism of action of these anti-inflammatory agents - reduction of inflammatory markers as body fat, gene expression and cytokine dosages, such as IL-1, IL-6, IL-8, TNF-alfa, leptin, resistin, inhibition of the enzyme cyclooxygenase (COX-2) and nitric oxide-synthase induced (iNOS), increased levels of adiponectin, reduction of expression of the enzyme 5-lipoxygenase (5-LOX), better insulin sensitivity, and PCR reduction.

### 2.5 Data synthesis and analysis

The extraction of all data will be standardized and performed by 2 independent authors (S.S.C.N., J.L.C.Q.), creating a database in a pre-designed and previously tested spreadsheet in the Excel program. The third author (A.F.M.) will verify the discrepancies and organize the information to construct the original text. The following information will be presented in this database: author and year of publication, type of study, number and type of control groups, species, sex, weight, age, oral diet, experiment time, an anti-inflammatory agent used and its mechanism of action, dosage, time of administration, frequency of administration, type of administration and vehicle, statistical measures used and results. If any data is not contained in the article, we will contact the authors requesting the information.

At the end of the studies data analysis, at least 2 studies, if clinical and/or methodological and/or statistical homogeneity are found, a meta-analysis will be performed. This will be carried out using the Rev Man Analyses statistical package in Review Manager v.5.3. If, however, the studies are considered too heterogeneous, only one narrative synthesis will be performed.

The random-effects model will be used to account for anticipated heterogeneity because of the exploratory nature of animal studies. For dichotomous outcomes, we will derive the OR and 95% CI for each study. The heterogeneity between the trial results will be evaluated using a standard *I*^2^ test with a significance level of *P* .1, and the *I*^2^ statistic, which is a quantitative measure of inconsistency across studies, with a value of 0% indicating no observed heterogeneity, and values of 50% showing substantial levels are present. If there is heterogeneity (*I*^2^ 75%), a random-effects model will be used to combine the trials to calculate the relative risk (RR) and 95% CI, using the DerSimonian-Laird algorithm in meta for a package, a meta-analysis package for R. If possible, funnel plots will also be used to assess the presence of potential reporting biases. A linear regression approach will be used to evaluate funnel plot asymmetry. Adjustments will be performed, if needed in the studies.

### 2.6 Subgroup analysis

Subgroup analyses will be performed if relevant data are available. If possible, the following will be undertaken: species (stratified per species); type of anti-inflammatory agent (stratified per agent); duration of treatment; dose; effect dose of anti-inflammatory agents; mechanism of action of anti-inflammatory agents.

### 2.7 Risk of bias and quality assessment

The Systematic Review Center for Laboratory Animal Experimentation (Syrcle) tool will be used to assess the risk of bias.

### 2.8 Ethics and dissemination

Ethical approval and informed consent are not necessary for this research because it is a systematic review (use of secondary data).

## 3 Discussion

The search for new pharmacological approaches for the treatment of obesity has been reported in the literature, as conventional drugs used are limited by the non-specificity and unsustainable adverse effects of weight loss.^[20]^ Most anti-obesity drugs work by suppressing appetite, increasing neurotransmitters, such as serotonin, norepinephrine, and dopamine, or inhibiting lipid absorption, such as serotonin, norepinephrine, and dopamine, or inhibiting lipid absorption.^[21]^ However, many of these drugs have been associated with an increase in cardiovascular events and severe side effects, such as pulmonary hypertension, stroke, psychotic behaviors, risk of dependence, and gastrointestinal side effects.^[20,22,23]^

Therefore, studies that address the treatment of obesity with nutraceutical agents such as anti-inflammatory drugs are very relevant, considering the growing number of people with this pathology and the various diseases triggered by adipose tissue accumulation. A systematic review study discussed the main mechanisms of action of polyphenols and fatty acids in the diet, evaluated separately and in combination. These compounds act as anti-inflammatory agents associated with adipose tissue. Several routes, including AMPK and the PPAR-γ, activate them. Besides, they may act by the suppression of receptors TLRs and via NF-κB.^[24]^

In another systematic review study, Sibuyi et al^[12]^ pointed anti-obesity strategies based on nanotechnology - using drugs, peptides, and bioactive compounds - aiming at their effect on white adipose tissue (WAT) and its vasculature to reverse obesity. These mechanisms, with strategies aimed at white adipose tissue, which act by reducing its size, destroying hypertrophic adipocytes, transforming WAT into brown adipose tissue (BAT), or inhibiting adipogenesis, may be ideal in the treatment of obesity and associated comorbidities. Compared to conventional therapy, they have a high tolerance and improved effectiveness associated with few side effects. The study shows the need to develop strategies for the treatment of obesity, targeting therapies mainly in adipose tissue, evaluating the mechanisms involved in the anti-inflammatory action of molecules on adipocytes.

Studies that address these mechanisms are still scarce in the literature, and it is crucial to produce articles that support new research on the topic. Thus, it is necessary to provide systematic reviews that focus on the anti-inflammatory agents’ mechanisms in adipose tissue, considering that the treatment directed to this tissue has shown to be promising when compared to traditional medicines. The present protocol will assist in the production of a systematic review identifying these mechanisms basing new research and, consequently, assisting in the development of new therapies used in humans.

## Author contributions

**Conceptualization:** Thaís Souza Passos, Ana Heloneida de Araújo morais.

**Data curation:** Sara Sayonara da Cruz Nascimento, Grasiela Piuvezam, Bruna Leal Lima Maciel, Thaís Souza Passos, Ana Heloneida de Araújo morais.

**Formal analysis:** Sara Sayonara da Cruz Nascimento, Grasiela Piuvezam, Bruna Leal Lima Maciel, Thaís Souza Passos, Ana Heloneida de Araújo morais.

**Funding acquisition:** Thaís Souza Passos, Ana Heloneida de Araújo morais.

**Investigation:** Sara Sayonara da Cruz Nascimento, Jaluza Luana Carvalho de Queiroz, Amanda Fernandes de Medeiros, Ana Clara de França Nunes, Grasiela Piuvezam, Thaís Souza Passos.

**Methodology:** Sara Sayonara da Cruz Nascimento, Jaluza Luana Carvalho de Queiroz, Amanda Fernandes de Medeiros, Ana Clara de França Nunes, Grasiela Piuvezam, Thaís Souza Passos, Ana Heloneida de Araújo morais.

**Project administration:** Grasiela Piuvezam, Thaís Souza Passos, Ana Heloneida de Araújo morais.

**Supervision:** Grasiela Piuvezam, Thaís Souza Passos, Ana Heloneida de Araújo morais.

**Validation:** Grasiela Piuvezam.

**Writing – original draft:** Sara Sayonara da Cruz Nascimento, Jaluza Luana Carvalho de Queiroz, Amanda Fernandes de Medeiros, Ana Clara de França Nunes, Grasiela Piuvezam.

**Writing – review & editing:** Sara Sayonara da Cruz Nascimento, Grasiela Piuvezam, Bruna Leal Lima Maciel, Thaís Souza Passos, Ana Heloneida de Araújo morais.

## References

[1] World Health Organization. Obesity and overweight: Fact sheet. WHO Media Centre. Published 2020. https://www.who.int/en/news-room/fact-sheets/detail/obesity-and-overweight. Accessed September 8, 2020.

[2] Kotzbeck P Giordano A Mondini E. Brown adipose tissue whitening leads to brown adipocyte death and adipose tissue inflammation. J Lipid Res 2018;59:784–94. 29599420 10.1194/jlr.M079665 PMC5928436

[3] Weisberg SP McCann D Desai M. Obesity is associated with macrophage accumulation in adipose tissue. J Clin Invest 2003;112:1796–808. 14679176 10.1172/JCI19246 PMC296995

[4] Xu H Barnes GT Yang Q. Chronic inflammation in fat plays a crucial role in the development of obesity-related insulin resistance. J Clin Invest 2003;112:1821–30. 14679177 10.1172/JCI19451 PMC296998

[5] Cinti S. The adipose organ at a glance. Dis Model Mech 2012;5:588–94. 22915020 10.1242/dmm.009662 PMC3424455

[6] Murano I Barbatelli G Parisani V. Dead adipocytes, detected as crown-like structures, are prevalent in visceral fat depots of genetically obese mice. J Lipid Res 2008;49:1562–8. 18390487 10.1194/jlr.M800019-JLR200

[7] Bartelt A Heeren J. Adipose tissue browning and metabolic health. Nat Rev Endocrinol 2014;10:24–36. 24146030 10.1038/nrendo.2013.204

[8] Drolet R Richard C Sniderman AD. Hypertrophy and hyperplasia of abdominal adipose tissues in women. Int J Obes 2008;32:283–91. 10.1038/sj.ijo.0803708 17726433

[9] Goossens GH. The metabolic phenotype in obesity: fat mass, body fat distribution, and adipose tissue function. Obes Facts 2017;doi:10.1159/000471488. 10.1159/000471488 PMC5644968 28564650

[10] Illesca P Valenzuela R Espinosa A. Hydroxytyrosol supplementation ameliorates the metabolic disturbances in white adipose tissue from mice fed a high-fat diet through recovery of transcription factors Nrf2, SREBP-1c, PPAR-( and NF-(B. Biomed Pharmacother 2019;109 (September 2018):2472–81. 30551508 10.1016/j.biopha.2018.11.120

[11] Luna-Vital D Luzardo-Ocampo I Cuellar-Nuñez ML. Maize extract rich in ferulic acid and anthocyanins prevents high-fat-induced obesity in mice by modulating SIRT1, AMPK and IL-6 associated metabolic and inflammatory pathways. J Nutr Biochem 2020;79:108343 http://www.sciencedirect.com/science/article/pii/S095528631930717X 32007662 10.1016/j.jnutbio.2020.108343

[12] Sibuyi NRS Moabelo KL Meyer M. Nanotechnology advances towards development of targeted-treatment for obesity. J Nanobiotechnology 2019;17:1–21. 31842876 10.1186/s12951-019-0554-3 PMC6913004

[13] Bray GA Greenway FL. Pharmacological treatment of the overweight patient. Pharmacol Rev 2007;59:151–84. 17540905 10.1124/pr.59.2.2

[14] El-Menshawe SF Ali AA Rabeh MA. Nanosized soy phytosome-based thermogel as topical anti-obesity formulation: An approach for acceptable level of evidence of an effective novel herbal weight loss product. Int J Nanomedicine 2018;13:307–18. 29391791 10.2147/IJN.S153429 PMC5768425

[15] Kesarwani K Gupta R. Bioavailability enhancers of herbal origin: an overview. Asian Pac J Trop Biomed 2013;3:253–66. 23620848 10.1016/S2221-1691(13)60060-X PMC3634921

[16] Jungbauer A Medjakovic S. Anti-inflammatory properties of culinary herbs and spices that ameliorate the effects of metabolic syndrome. Maturitas 2012;71:227–39. 22226987 10.1016/j.maturitas.2011.12.009

[17] Ebrahimzadeh Attari V Malek Mahdavi A Javadivala Z. A systematic review of the anti-obesity and weight lowering effect of ginger (Zingiber officinale Roscoe) and its mechanisms of action. Phyther Res 2018;32: doi:10.1002/ptr.5986. 10.1002/ptr.5986 29193411

[18] Ouzzani M Hammady H Fedorowicz Z. Rayyan-a web and mobile app for systematic reviews. Syst Rev 2016;5:1–0. 27919275 10.1186/s13643-016-0384-4 PMC5139140

[19] Zaugg H West RE Tateishi I. Mendeley: creating communities of scholarly inquiry through research collaboration. Tech Trends 2011;55:32–6.

[20] Bray GA Heisel WE Afshin A. The science of obesity management: an endocrine society scientific statement. Endocr Rev 2018;39:79–132. 29518206 10.1210/er.2017-00253 PMC5888222

[21] James WPT. The epidemiology of obesity: the size of the problem. J Intern Med 2008;263:336–52. 18312311 10.1111/j.1365-2796.2008.01922.x

[22] Padwal RS Majumdar SR. Drug treatments for obesity: orlistat, sibutramine, and rimonabant. Lancet 2007;369:71–7. 17208644 10.1016/S0140-6736(07)60033-6

[23] Kang JG Park CY. Anti-obesity drugs: a review about their effects and safety. Diabetes Metab J 2012;36:13–25. 22363917 10.4093/dmj.2012.36.1.13 PMC3283822

[24] Siriwardhana N Kalupahana NS Cekanova M. Modulation of adipose tissue inflammation by bioactive food compounds. J Nutr Biochem 2013;24:613–23. 23498665 10.1016/j.jnutbio.2012.12.013

